# Barriers and drivers influencing people's behaviour towards COVID-19 public health and social measures in the Netherlands

**DOI:** 10.1016/j.puhip.2024.100566

**Published:** 2024-12-19

**Authors:** Valérie Eijrond, Nora Bünemann, Nicky Renna, Brett Craig, Katrine Bach Habersaat, Hélène Voeten, Pearl Dykstra, Anja Schreijer

**Affiliations:** a Erasmus MC, Pandemic and Disaster Preparedness Center, Delft, Rotterdam, the Netherlands; b Erasmus University Rotterdam, Pandemic and Disaster Preparedness Center, Delft, Rotterdam, the Netherlands; c Erasmus University Rotterdam, Department of Public Administration and Sociology, Rotterdam, the Netherlands; d WHO Regional Office for Europe, Copenhagen, Denmark; e Public Health Service (GGD) Rotterdam-Rijnmond, Rotterdam, the Netherlands; f Department of Public Health, Erasmus MC, Rotterdam, the Netherlands

**Keywords:** Tailoring health programmes, Underserved groups, COVID-19, Behavioural insights, Public health and social measures, Health inequities

## Abstract

**Background:**

The disease burden of COVID-19 infection, morbidity, and mortality was unevenly distributed across different population subgroups. A one-size-fits-all approach may not reach all groups. Identifying barriers and drivers that influence behaviour towards COVID-19 public health and social measures (PHSM) is an important step when designing tailored interventions. Using the WHO Tailoring Health Programmes (THP) approach, we performed a situation analysis. The objectives were to identify population subgroups; gain an overview of barriers and drivers to COVID-19 PHSM among subgroups; and interventions and research initiated in the Netherlands.

**Study design:**

A literature scan, interviews and a meeting with experts were held.

**Methods:**

Results were categorised according to the capability, opportunity, and motivation to understand COVID-19 PHSM behaviour.

**Results:**

Different population subgroups have been studied regarding their barriers and drivers for uptake and adherence to COVID-19 PHSM, such as (older) migrant communities. Barriers include language barriers, mis- and disinformation and distrust. Drivers include protecting others and oneself. Network ties play a role, as a barrier and driver. Forty-five interventions and research projects were identified. Several revealed the importance of involving key figures. A lack of monitoring and evaluation of interventions during the pandemic was acknowledged by experts.

**Conclusions:**

The situation analysis reveals that knowledge of the most prevalent barriers and drivers between underserved groups and how to address them with targeted (cost) effective interventions is lacking. With this THP project we aim to develop new or improve existing interventions addressing behaviours towards PHSM among a prioritised population group informed by evidence-based behavioural insights.

## 1 Introduction

The disease burden of COVID-19 infection, morbidity, and mortality was unevenly distributed across different population groups in the Netherlands. Furthermore, the COVID-19 vaccination acceptance^1^ and uptake^2^ exhibit disparities among groups [1]. During the vaccination campaigns, acceptance was lower among young adults, people with a migrant background, and people with a lower education level [1]. A possible explanation is that public health and social measures (PHSM),^3^ including vaccination, testing and other measures, e.g., home isolation, were not aligned to the needs and circumstances of people and communities [2]. A one-size-fits-all approach risks excluding population subgroups, suggesting there is a need for targeted interventions that reflect the beliefs and needs of these groups [1,3]. Identifying barriers and drivers for specific groups towards COVID-19 PHSM is a crucial step in designing tailored interventions.

The World Health Organization (WHO) Tailoring Health Programmes (THP) approach serves as a diagnostic guide, aimed at identifying population subgroups with a suboptimal uptake of a health behaviour, as well as capturing the related barriers and drivers. These insights are used to develop tailored interventions, contributing to improved health outcomes, and address health inequities [2]. The aim of this short communication is to report the findings of the first phase of the THP approach: the situation analysis.^4^ The objective is threefold.

1) to identify the population subgroups with a lower uptake and adherence to COVID-19 PHSM;
2) to gain an overview of previously identified barriers and drivers of the general population and population subgroups to COVID-19 PHSM; and
3) to obtain an overview of interventions and research initiated by various stakeholders in the Netherlands.

## 2 Methods

A literature scan of peer-reviewed and grey literature was conducted (see Supplementary Materials). In total 58 articles were included. To complement the literature review, interviews were held with 23 experts, including policy makers (n = 5), social and behavioural sciences researchers (n = 12) and (bio)medical specialists (n = 6) during August and September 2022. Subsequently, an expert meeting took place on October 11, 2022 in the Netherlands, facilitated by the Pandemic and Disaster Preparedness Center and the WHO Regional Office for Europe. The meeting was attended by 16 representatives from academia, government, and municipal health services. The objectives were to present and discuss the preliminary findings of the situation analysis and to discuss the requirements to develop, test and evaluate tailored interventions for population subgroups with a lower uptake and adherence to COVID-19 PHSM.

An adapted capability-opportunity-motivation-behaviour (COM-B) model [2,7] provided the theoretical framework for categorising the barriers and drivers. The model delineates three inter-linked factors: capability and motivation at the individual level and opportunity within the contextual realm, which are essential for any health behaviour to occur [8].

## 3 Results

### 3.1 Identified population subgroups

Based on the literature, multiple population subgroups have been studied regarding their barriers and drivers for their uptake and adherence to COVID-19 PHSM (see Supplementary Materials). These include (older) migrant communities (i.e. Moroccan and Turkish), homeless people, youth/young adults (aged 12–30), people with a low socioeconomic status (SES) or living in low SES neighbourhoods and people with low health literacy. In addition to the above-mentioned groups, experts also mentioned the following groups: sex workers, people prone to conspiracy theories, asylum seekers, status holders,^5^ and people with disabilities.

### 3.2 Identified barriers and drivers to COVID-19 PHSM

**Fig. 1:**
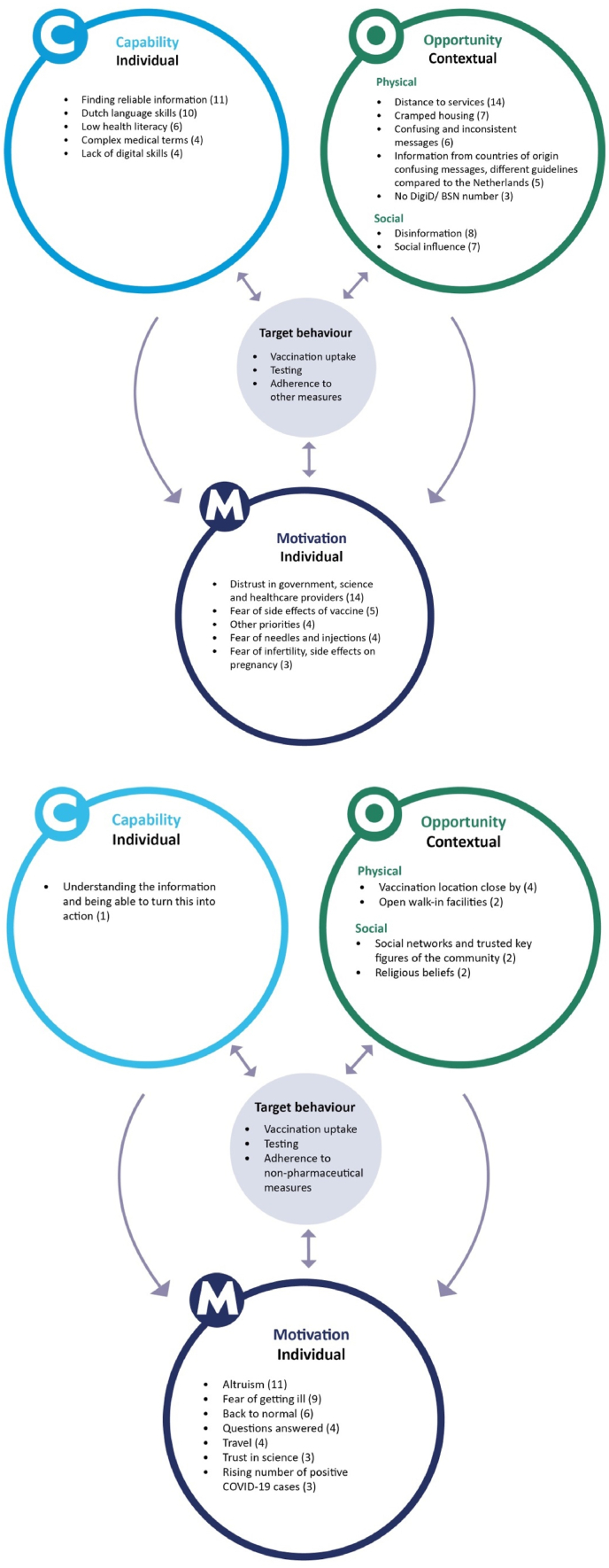
Overview of the barriers (left) and drivers (right) influencing adherence to COVID-19 public health and social measures most often mentioned by interviewed experts (n = 23), by COM-B factors [7].

Multifactorial barriers and drivers were identified (see Fig. 1 and Supplementary Materials), with similarities and differences between the general population and subgroups. Identified barriers across groups included lack of trust in the government, issues related to information (i.e. insufficient, complex, contradictory, and misinformation) and low risk perception and severity of the disease. For vaccination, vaccine safety and the short- and long-term side effects emerged as barriers. Drivers across groups included self-protection, the moral duty to protect others, and the reopening of society. Experts also mentioned some barriers that were more prevalent among some subgroups. Among migrants and refugees, these included lack of a social security number (DigID/)BSN and problems with Dutch language skills. Distance to vaccination and test locations were a barrier among older people, people with a migrant background and people with a low SES. For the adherence to other measures, the inability to work from home due to occupation type or cramped housing conditions were identified as a barrier. Network ties may act as a driver and barrier. The desire to protect one's family and friends from infection and severe illness can be a driver to vaccinate. Conversely, fear of stigmatisation from family and friends can act as a barrier. The majority of existing research is quantitative and has predominantly focused on understanding (vaccination) behaviour among the general population, rather than in subgroups. Moreover, there was a notably reduced emphasis on identifying drivers compared to barriers, most likely because these were not studied within subgroups rather than being absent. This is supported by the results of the interviews with experts in which more barriers than drivers were highlighted.

### 3.3 Interventions and research projects

Over 45 interventions and research projects related to COVID-19 PHSM were identified. The three highlighted interventions - tailored vaccination and education, risk-oriented large-scale testing and the vaccination doubt line^6^ - addressed capability, physical, social and motivational barriers. Interventions were implemented on a local, regional and national level. Experts noted that key figures, such as general practitioners and trusted religious leaders, played an important role in identifying groups with a lower acceptance or uptake and in the design and implementation of interventions. The participating experts concluded that there was a lack of monitoring and evaluation of interventions during the pandemic, due to continuous changes, time constraints and lack of resources. Consequently, knowledge gaps remain regarding the (cost)-effectiveness of interventions and which interventions have proven successful for specific subgroups.

Based on the situation analysis, our results identified the population subgroups that are so-called underserved [2,8], such as migrant communities and older people. Underserved groups have a lower acceptance or uptake to get vaccinated, tested or adhere to other measures. They may encounter distinct barriers and drivers compared to the general population, face barriers accessing healthcare services and were possibly not reached through national campaigns and public health strategies.

## 4 Discussion

This situation analysis marks the initial phase of the THP approach. To our knowledge, existing Dutch literature lacks research focused on certain groups, including disabled people, refugees (e.g., Syrians) and migrant communities (e.g., Indonesians). More specifically, research into the multifaceted barriers and especially drivers of various underserved groups is missing. Further research is needed to study the role of family members, caregivers and key figures as sources of information and as shapers of vaccination/testing behaviour (see also [10]).

Multi-method research is necessary to acquire a rich understanding of a target group's perspective and to determine the most important determinants of behaviour across a larger representative sample. When developing cost-effective interventions, it is valuable to differentiate between the most prevalent and important barriers and drivers among different underserved groups, as some may be more pronounced in some groups. Moreover, keep in mind that a multitude of barriers may influence people's decision to vaccinate. For instance, even if vaccination becomes more accessible, some people might still choose not to get vaccinated, due to ongoing concerns about vaccination safety or government trust. Hence, it's crucial to adopt a multi-level approach that addresses multiple barriers within interventions. The results of the situation analysis suggest that the barriers disinformation and distrust towards the government, institutions and vaccines, demand attention on how to tackle them.

## 5 Conclusion

The pandemic shined a spotlight on the health disparities and unmet needs of underserved groups. This calls for the use of behavioural insights to tailor interventions according to the needs and circumstances of underserved groups. Our situation analysis highlights the absence of comprehensive knowledge regarding the most prevalent barriers and drivers among underserved groups in the Netherlands and how to effectively address them. Although the highlighted interventions demonstrate promising examples of employing behavioural insights to tackle health disparities, there was insufficient monitoring and evaluation of these interventions to ascertain their (cost) effectiveness. This paper can inform scoping and problem formulation for the next step of the THP approach: conducting primary research into the barriers and drivers towards PHSM among a prioritised population group.

## Authors’ contributions

All authors read and approved the final version of the manuscript.

## Disclaimer

The authors affiliated with the World Health Organization (WHO) are alone responsible for the views expressed in this publication and they do not necessarily represent the decisions or policies of the WHO.

## Funding

This research did not receive any specific grant from funding agencies in the public, commercial, or not-for-profit sectors.

## Declaration of competing interest

The authors declare that they have no known competing financial interests or personal relationships that could have appeared to influence the work reported in this paper.

## Footnotes

1 Vaccination acceptance: The degree to which individuals accept, question or refuse vaccination [4].

2 Vaccination uptake: The use of a vaccine in an immunisation program [4].

3 ‘Public health and social measures (PHSM): Actions or measures taken by individuals, institutions, communities, local and national governments to reduce the spread of COVID-19. PHSM include non-pharmaceutical interventions, physical distancing measures, pre- and post-exposure prophylaxis and vaccines.’ [5].

4 See full report: Pandemic and Disaster Preparedness Center (2023). One size does not fit all! Barriers and drivers influencing towards people's behaviour towards COVID-19 public health and social measures in the Netherlands. Rotterdam. Retrieved from: https://convergence.nl/one-size-does-not-fit-all/ [6].

5 Recognised asylum seekers who have a residence permit and are allowed to study/work in the Netherlands.

6 The goal of vaccination doubt line was to offer individualised information and medical guidance regarding the COVID-19 vaccines [9].
