# Supplementary material for: Barriers and drivers influencing people's behaviour towards COVID-19 public health and social measures in the Netherlands
Source: Public Health Pract (Oxf). 2024 Dec 19;9:100566. doi: 10.1016/j.puhip.2024.100566 (PMC11732217; doi:10.1016/j.puhip.2024.100566)
Supplement: Multimedia component 1 [file mmc1.docx]

The search was performed in Embase, Medline and Web of Science on the 13th of July 2022. Articles were included if they were published between 2019 and 2022 using data gathered in the Netherlands, and if they focused on COVID-19 and vaccine acceptance and uptake, testing, compliance to non-pharmaceutical measures, beliefs, trust, attitudes, concerns, perceptions, barriers and drivers. The literature was supplemented according to the snowball method, : in which reference lists of included articles were checked to retrieve additional relevant articles. Other websites were searched for additional relevant documents in English as well as in Dutch, including grey literature, pre-prints, working papers, memos and projects from international literature. The literature was complemented with (unpublished) articles, presentations and reports received by the interviewed experts.

**Search terms:**

| **Database searched** | **Platform** | **Years of coverage** | **Records** | **Records after duplicates removed** |
| --- | --- | --- | --- | --- |
| Embase | Embase.com | 1971 - Present | 74 | 71 |
| Medline ALL | Ovid | 1946 - Present | 46 | 14 |
| Web of Science Core Collection* | Web of Knowledge | 1975 - Present | 36 | 5 |
| **Total** | | | **156** | **90** |

*Science Citation Index Expanded (1975-present) ; Social Sciences Citation Index (1975-present) ; Arts & Humanities Citation Index (1975-present) ; Conference Proceedings Citation Index- Science (1990-present) ; Conference Proceedings Citation Index- Social Science & Humanities (1990-present) ; Emerging Sources Citation Index (2005-present)

**Netherlands**

**embase.com 74**

('SARS-CoV-2 vaccine'/exp OR 'COVID-19 testing'/exp OR (('coronavirus disease 2019'/de OR pandemic/de) AND ('social distancing'/de OR 'face mask'/de)) OR (((SARS-CoV-2 OR SARSCoV2 OR covid* OR corona* OR pandemic*) NEAR/3 (vaccin*))):ab,ti OR (((SARS-CoV-2 OR SARSCoV2 OR covid* OR corona*) AND (vaccin* OR booster* OR test*))):ti) AND (motivation/exp OR willingness/de OR anxiety/exp OR attitude/exp OR fear/de OR 'refusal to participate'/de OR 'public opinion'/de OR 'treatment refusal'/de OR prejudice/de OR (motivat* OR willing* OR anxiety OR attitude* OR belief* OR fear* OR distrust* OR mistrust* OR refus* OR reject* OR public-opinion* OR ((vaccin* OR booster* OR test* OR social-distanc* OR social-isolat* OR physical-distanc* OR face-mask* OR facemask* OR government-measures* OR prevent*-measures* OR protect*-measures* OR restrict*-measures* OR lockdown* OR lock-down* OR government*-health-measures* OR containment-measures*) NEAR/3 (awareness OR behavior OR behaviour OR hesitanc* OR trust OR criticis* OR doubt* OR dropout* OR exemption* OR perception OR rumor* OR rumour* OR intent* OR controvers* OR misconception* OR misinformation OR opposition OR delay OR dilemma* OR objector* OR resist* OR sceptic OR uptake OR barrier* OR choice* OR concern* OR accepta* OR complian* OR adher* OR noncomplian* OR nonadher*)) OR anti-vaccin* or antivaccin* OR under-vaccin* or undervaccin*):Ab,ti OR (determin* OR correlate* OR demograph* OR sociodemograph* OR heterogen* OR disparit* OR inequit* OR driver*):ti) AND (Netherlands/exp OR Benelux/de OR (Netherlands OR dutch OR Benelux):ab,ti)

**Medline ALL Ovid 46**

(exp COVID-19 Vaccines / OR exp COVID-19 Testing / OR ((COVID-19/ OR Pandemics/) AND (Physical Distancing/)) OR (((SARS-CoV-2 OR SARSCoV2 OR covid* OR corona* OR pandemic*) ADJ3 (vaccin*))).ab,ti. OR (((SARS-CoV-2 OR SARSCoV2 OR covid* OR corona*) AND (vaccin* OR booster* OR test*))).ti.) AND (Motivation / OR exp Anxiety / OR exp Attitude / OR exp Fear / OR exp Refusal to Participate / OR Public Opinion / OR exp Treatment Refusal / OR Prejudice / OR (motivat* OR willing* OR anxiety OR attitude* OR belief* OR fear* OR distrust* OR mistrust* OR refus* OR reject* OR public-opinion* OR ((vaccin* OR booster* OR test* OR social-distanc* OR social-isolat* OR physical-distanc* OR face-mask* OR facemask* OR government-measures* OR prevent*-measures* OR protect*-measures* OR restrict*-measures* OR lockdown* OR lock-down* OR government*-health-measures* OR containment-measures*) ADJ3 (awareness OR behavior OR behaviour OR hesitanc* OR trust OR criticis* OR doubt* OR dropout* OR exemption* OR perception OR rumor* OR rumour* OR intent* OR controvers* OR misconception* OR misinformation OR opposition OR delay OR dilemma* OR objector* OR resist* OR sceptic OR uptake OR barrier* OR choice* OR concern* OR accepta* OR complian* OR adher* OR noncomplian* OR nonadher*)) OR anti-vaccin* or antivaccin* OR under-vaccin* or undervaccin*).ab,ti. OR (determin* OR correlate* OR demograph* OR sociodemograph* OR heterogen* OR disparit* OR inequit* OR driver*).ti.) AND (Netherlands / OR (Netherlands OR dutch OR Benelux).ab,ti.)

**Web of science 36**

(TS=(((SARS-CoV-2 OR SARSCoV2 OR covid* OR corona* OR pandemic*) NEAR/2 (vaccin*))) OR TI=(((SARS-CoV-2 OR SARSCoV2 OR covid* OR corona*) AND (vaccin* OR booster* OR test*)))) AND (TS=(motivat* OR willing* OR anxiety OR attitude* OR belief* OR fear* OR distrust* OR mistrust* OR refus* OR reject* OR public-opinion* OR ((vaccin* OR booster* OR test* OR social-distanc* OR social-isolat* OR physical-distanc* OR face-mask* OR facemask* OR government-measures* OR prevent*-measures* OR protect*-measures* OR restrict*-measures* OR lockdown* OR lock-down* OR government*-health-measures* OR containment-measures*) NEAR/2 (awareness OR behavior OR behaviour OR hesitanc* OR trust OR criticis* OR doubt* OR dropout* OR exemption* OR perception OR rumor* OR rumour* OR intent* OR controvers* OR misconception* OR misinformation OR opposition OR delay OR dilemma* OR objector* OR resist* OR sceptic OR uptake OR barrier* OR choice* OR concern* OR accepta* OR complian* OR adher* OR noncomplian* OR nonadher*)) OR anti-vaccin* or antivaccin* OR under-vaccin* or undervaccin*) OR TI=(determin* OR correlate* OR demograph* OR sociodemograph* OR heterogen* OR disparit* OR inequit* OR driver*)) AND TS=((Netherlands OR dutch OR Benelux))
