# Supplementary material for: Barriers and drivers influencing people's behaviour towards COVID-19 public health and social measures in the Netherlands
Source: Public Health Pract (Oxf). 2024 Dec 19;9:100566. doi: 10.1016/j.puhip.2024.100566 (PMC11732217; doi:10.1016/j.puhip.2024.100566)
Supplement: Multimedia component 2 [file mmc2.docx]

Table 1: Barriers towards COVID-19 vaccination across different population subgroups

| **Behaviour:**  **VACCINATION** |  | **Population subgroups** | | | | | | |
| --- | --- | --- | --- | --- | --- | --- | --- | --- |
|  |  | General  Population^[[1]](#footnote-1)^ | Youth/ Young  Adults^[[2]](#footnote-2)^ | People with  a migration background ^[[3]](#footnote-3)^ | Low SES^[[4]](#footnote-4)^ | Older Migrants^[[5]](#footnote-5)^ | Homeless  People^[[6]](#footnote-6)^ | Low Health  Skills^[[7]](#footnote-7)^ |
| **COM-B** | **Barriers** |  |  |  |  |  |  |  |
| **Capability**  **(individual)** |  | | | | | | | |
|  | Language barriers |  |  |  |  |  |  |  |
|  | Difficulty making an (online) appointment (digital skills) |  |  |  |  |  |  |  |
|  | No transport to inaccessible vaccination location |  |  |  |  |  |  |  |
| **Opportunity (contextual)** |  | | | | | | | |
| **Societal** | Lack of obligations: No pressure and obligation from the government |  |  |  |  |  |  |  |
|  | Famous Dutch people who do not vaccinate |  |  |  |  |  |  |  |
|  | Religious beliefs |  |  |  |  |  |  |  |
|  | Influence of religious and political leaders |  |  |  |  |  |  |  |
|  | Strong influence of the social environment |  |  |  |  |  |  |  |
|  | Social pressure |  |  |  |  |  |  |  |
|  | Social norms, following trusted friends or leaders in a community |  |  |  |  |  |  |  |
|  | Many rumours from various sources about adverse effects |  |  |  |  |  |  |  |
|  | If I get sick, this is predestined (lies in God's hands) |  |  |  |  |  |  |  |
|  | Misinformation |  |  |  |  |  |  |  |
| **Physical (information)** | Information needed about side effects among people with the same characteristics (age) or comorbidities as themselves |  |  |  |  |  |  |  |
|  | Contradictory information |  |  |  |  |  |  |  |
|  | Insufficient information about vaccines |  |  |  |  |  |  |  |
|  | (Fear & uncertainty) from unreliable information |  |  |  |  |  |  |  |

|  |  | **Population subgroups** | | | | | | |
| --- | --- | --- | --- | --- | --- | --- | --- | --- |
|  |  | General  Population | Youth/ Young  Adults | People with  a migration background | Low SES | Older Migrants | Homeless  People | Low Health  Skills |
|  | Opposing messages because information from the own community or home country does not correspond with information from te Dutch government |  |  |  |  |  |  |  |
|  | Unilateral reporting by government and media |  |  |  |  |  |  |  |
| **Motivation (individual)** |  | | | | | | | |
| **Attitudes, perception, risk assessment** | Low sense of urgency |  |  |  |  |  |  |  |
| **Attitudes, perception, risk assessment** | Low perceived risk and severity of disease |  |  |  |  |  |  |  |
|  | I don't need it, I'm healthy enough |  |  |  |  |  |  |  |
|  | I do not believe that COVID-19 poses a serious threat (to me and public health) |  |  |  |  |  |  |  |
|  | I have already had COVID-19 |  |  |  |  |  |  |  |
|  | Sufficient protection from the other vaccinations |  |  |  |  |  |  |  |
|  | Not a risk group yourself / Believe in your own immune system |  |  |  |  |  |  |  |
|  | COVID-19 is not a serious disease |  |  |  |  |  |  |  |
|  | Have been infected before, don't expect to get infected again |  |  |  |  |  |  |  |
|  | Expectation that herd immunity will take place |  |  |  |  |  |  |  |
|  | Sceptical attitude versus vaccines |  |  |  |  |  |  |  |
| **Trust (in vaccines)** | Concerns about short-term and long-term side effects / that vaccines are harmful **or** have side effects |  |  |  |  |  |  |  |
| **Trust (in vaccines)** | Side effects of previous injection(s) |  |  |  |  |  |  |  |
|  | Doubts about the effectiveness of the vaccine |  |  |  |  |  |  |  |
|  | Doubts/concerns about vaccine safety |  |  |  |  |  |  |  |
|  | Concerns about the side effects of the vaccine in relation to their specific medical condition |  |  |  |  |  |  |  |
|  | Distrust mRNA new vaccines |  |  |  |  |  |  |  |
|  | Doubt about certain types of vaccines |  |  |  |  |  |  |  |
|  | Consider vaccines unnecessary |  |  |  |  |  |  |  |

|  |  | **Population subgroups** | | | | | | |
| --- | --- | --- | --- | --- | --- | --- | --- | --- |
|  |  | General  Population | Youth/ Young  Adults | People with  a migration background | Low SES | Older Migrants | Homeless  People | Low Health  Skills |
|  | It is better to become immune to COVID-19 through infection than through a vaccine |  |  |  |  |  |  |  |
|  | I do not want to take a vaccine if there is a chance that you will still be infected with COVID-19 |  |  |  |  |  |  |  |
|  | Two vaccinations are more than enough |  |  |  |  |  |  |  |
|  | Waiting because quality of the vaccine will improve over time |  |  |  |  |  |  |  |
|  | Too early to see the negative effects |  |  |  |  |  |  |  |
|  | Do not know what is in the vaccine |  |  |  |  |  |  |  |
|  | Lead to other complications (e.g., heart attack, death) |  |  |  |  |  |  |  |
|  | Concerns about (in)fertility |  |  |  |  |  |  |  |
|  | Concerns vaccine discovered too quickly |  |  |  |  |  |  |  |
|  | Fear of the unknown |  |  |  |  |  |  |  |
|  | No trust in the vaccine |  |  |  |  |  |  |  |
|  | High speed development of vaccines |  |  |  |  |  |  |  |
|  | Vaccine is still in a testing phase |  |  |  |  |  |  |  |
|  | Generalised lack of governmental trust |  |  |  |  |  |  |  |
|  | Distrust in the pharma industry |  |  |  |  |  |  |  |
| **Trust (in institutions)** | Experiencing pressure/pressure from the government to vaccinate |  |  |  |  |  |  |  |
| **Trust (in institutions)**  **Other** | Lack of trust in the media |  |  |  |  |  |  |  |
|  | GGD is unreliable |  |  |  |  |  |  |  |
|  | Inserting a chip |  |  |  |  |  |  |  |
|  | Resistance: protecting the integrity of one's own body in response to restrictions of freedom/coercion/ ‘being pressured’ |  |  |  |  |  |  |  |
|  | Increasing/high vaccination rate works as a barrier (free-riding). If already high, ‘I no longer need to do it’ |  |  |  |  |  |  |  |
|  | Wait-and-see attitude 1: more knowledge (regarding long-term consequences and effectiveness) could give them greater confidence in the vaccine and could increase acceptance to be vaccinated |  |  |  |  |  |  |  |

|  |  | **Population subgroups** | | | | | | |
| --- | --- | --- | --- | --- | --- | --- | --- | --- |
|  |  | General  Population | Youth/ Young  Adults | People with  a migration background | Low SES | Older Migrants | Homeless  People | Low Health  Skills |
| **Other** | Wait-and-see attitude 2: want to make their own assessment, with the reason that they expect that the usefulness of vaccination in the autumn will be greater than vaccination now; and that if they vaccinate now, the vaccines will no longer work sufficiently in the autumn |  |  |  |  |  |  |  |
|  | Fear for needles |  |  |  |  |  |  |  |
|  | Doubt from believing in conspiracy theories |  |  |  |  |  |  |  |
|  | Negative experiences with the flu shot |  |  |  |  |  |  |  |
|  | Not thinking about/being involved with (the importance of) vaccination because there are more urgent problems in daily life (such as poverty) |  |  |  |  |  |  |  |
|  | Vaccinating is of no benefit to me |  |  |  |  |  |  |  |
|  | Production of antibodies after infection |  |  |  |  |  |  |  |
|  | The assumption that vaccination is only useful in case of complaints |  |  |  |  |  |  |  |
|  | Resistance to injecting something in the body |  |  |  |  |  |  |  |

Table 2: Drivers towards COVID-19 vaccination across different population subgroups

| **Behaviour:**  **VACCINATION** |  | **Population subgroups** | | | | |
| --- | --- | --- | --- | --- | --- | --- |
|  |  | General  Population^[[8]](#footnote-8)^ | Youth/ Young  Adults^[[9]](#footnote-9)^ | People with a migration background ^[[10]](#footnote-10)^ | Older Migrants^[[11]](#footnote-11)^ | Homeless  People^[[12]](#footnote-12)^ |
| **COM-B** | **Drivers** |  |  |  |  |  |
| **Capability (individual)** |  | | | | | |
|  | No values | No values | No values | No values | No values | No values |
| **Opportunity (contextual)** |  | | | | | |
| **Physical** | Cue to action: invitation letter or through trusted messages |  |  |  |  |  |
|  | Vaccinate on location |  |  |  |  |  |
|  | Vaccine passports made mandatory by the government |  |  |  |  |  |
|  | Trusted locations |  |  |  |  |  |
|  | Vaccination location nearby |  |  |  |  |  |
|  | Vaccination without appointment |  |  |  |  |  |
|  | Travel options |  |  |  |  |  |
|  | The use of role models |  |  |  |  |  |
|  | A personal approach by trusted care providers |  |  |  |  |  |
| **Social** | An increasing/high vaccination rate works as a driver (greater confidence in safety/effectiveness) |  |  |  |  |  |
|  | Social influence: behaviour and norms of family and friends. People around me do it / think it's important. Social norms (e.g., health care providers, people in their surroundings, public figures or important leaders) |  |  |  |  |  |
|  | Vaccination is key to reopening society: “COVID-19 crisis will only end if many people get vaccinated” |  |  |  |  |  |
|  | Engage in more social contacts and hug people, or take part in more activities again in free time |  |  |  |  |  |
|  | If freedoms for unvaccinated people are further restricted |  |  |  |  |  |
|  | Fighting the pandemic |  |  |  |  |  |

|  |  | **Population subgroups** | | | | |
| --- | --- | --- | --- | --- | --- | --- |
|  |  | General  Population | Youth/ Young  Adults | People with a migration background | Older Migrants | Homeless  People |
|  | Pressure from family |  |  |  |  |  |
|  | Peers' acceptance to vaccinate |  |  |  |  |  |
|  | Parents' expectations |  |  |  |  |  |
|  | Importance for the economy and society |  |  |  |  |  |
|  | Community involved |  |  |  |  |  |
|  | Collaboration with local care organisations |  |  |  |  |  |
| **Motivation (individual)** |  | | | | | |
| **Trust** | Trust in the government |  |  |  |  |  |
|  | Trust in science |  |  |  |  |  |
|  | Follow advice of their physicians |  |  |  |  |  |
|  | Rely on the safety of the vaccine |  |  |  |  |  |
| **Attitudes, perceptions, risk assessment** | Protect oneself |  |  |  |  |  |
|  | Sense of urgency: increasing hospital or intensive care (ICU) admissions |  |  |  |  |  |
|  | Perceived severity of the disease: as people would get more serious to get sick themselves or infect others, they are more willing to be vaccinated |  |  |  |  |  |
|  | (Changes in) personal health condition (i.e. age, risk group) |  |  |  |  |  |
|  | Belief that COVID-19 infection can be prevented through vaccination |  |  |  |  |  |
|  | Concerns about COVID-19 |  |  |  |  |  |
|  | Due to the restrictions in their social life (e.g., QR codes) |  |  |  |  |  |
|  | Instead of getting a PCR test all the time |  |  |  |  |  |
|  | Moral duty: sense of responsibility towards society |  |  |  |  |  |
|  | Heightened protection of family and friends |  |  |  |  |  |

Table 3: Barriers towards COVID-19 testing across different population subgroups

| **BEHAVIOUR: TESTING** |  | **Population subgroups** | | | | | | |
| --- | --- | --- | --- | --- | --- | --- | --- | --- |
|  |  | General  Population^[[13]](#footnote-13)^ | People with a migration background ^[[14]](#footnote-14)^ | Low Health Literacy^[[15]](#footnote-15)^ | Undocumented Migrants and Status Holders^[[16]](#footnote-16)^ | Undocumented Migrant Workers^[[17]](#footnote-17)^ | Older Migrants^[[18]](#footnote-18)^ | Low SES^[[19]](#footnote-19)^ |
| **COM-B** | **Barriers** |  |  |  |  |  |  |  |
| **Capability (individual)** |  | | | | | | | |
|  | Finding reliable information |  |  |  |  |  |  |  |
|  | Language barrier |  |  |  |  |  |  |  |
|  | No transportation |  |  |  |  |  |  |  |
|  | Applying for a COVID-19 test difficult |  |  |  |  |  |  |  |
| **Opportunity (contextual)** |  | | | | | | | |
|  | Lack of information on test locations |  |  |  |  |  |  |  |
|  | High costs of a self-test |  |  |  |  |  |  |  |
|  | Large distance to test locations |  |  |  |  |  |  |  |
|  | Long time to get a test (result) |  |  |  |  |  |  |  |
|  | Limited available times for setting an appointment |  |  |  |  |  |  |  |
|  | Inaccessibility of test streets |  |  |  |  |  |  |  |
|  | No BSN |  |  |  |  |  |  |  |
| **Social** | Taboo & stigmatization |  |  |  |  |  |  |  |
| **Motivation (individual)** |  | | | | | | | |
|  | Low trust in government (affiliated agencies) |  |  |  |  |  |  |  |
|  | Untrustworthiness test |  |  |  |  |  |  |  |
|  | Low risk perception |  |  |  |  |  |  |  |

|  |  | **Population subgroups** | | | | | | |
| --- | --- | --- | --- | --- | --- | --- | --- | --- |
|  |  | General  Population | People with a migration background | Low Health Literacy | Undocumented Migrants and Status Holders | Undocumented Migrant Workers | Older Migrants | Low SES |
|  | Belief that COVID-19 is not a serious disease |  |  |  |  |  |  |  |
|  | Mild complaints (e.g. runny nose) or have during the winter season |  |  |  |  |  |  |  |
|  | Complaints attributed to underlying conditions (e.g. asthma, hay fever) |  |  |  |  |  |  |  |
|  | Testing is unpleasant |  |  |  |  |  |  |  |
|  | Vaccinated against COVID-19 |  |  |  |  |  |  |  |
|  | Been infected before, so testing is pointless |  |  |  |  |  |  |  |
|  | Belief testing will yield nothing (it doesn’t make me better) |  |  |  |  |  |  |  |
|  | Fear request BSN, fear of being evicted |  |  |  |  |  |  |  |

Table 4: Drivers towards COVID-19 testing across different population subgroups

| **BEHAVIOUR: TESTING** |  | **Population subgroups** | | |
| --- | --- | --- | --- | --- |
|  |  | General  Population^[[20]](#footnote-20)^ | Undocumented Migrant Workers^[[21]](#footnote-21)^ | Low SES^[[22]](#footnote-22)^ |
| **COM-B** | **Drivers** |  |  |  |
| **Capability (individual)** |  | | | |
|  | No values | No values | No values | No values |
| **Opportunity (contextual)** |  | | | |
| **Physical** | Test location nearby |  |  |  |
|  | Testing without appointment |  |  |  |
|  | Low-threshold test methods |  |  |  |
|  | Free self-tests |  |  |  |
| **Motivation (individual)** |  | | | |
|  | Severe COVID-19-related complaints (e.g. fever, severe coughing) |  |  |  |
|  | Reassurance no COVID-19 |  |  |  |
|  | To be able to go (back) to work |  |  |  |
|  | Negative test certificate to gain access to cultural, social activities/ events or to go abroad |  |  |  |
|  | Not infecting others/ protect others |  |  |  |
|  | Response efficacy: See the added value of testing for themselves, others & the pandemic |  |  |  |
|  | Self-efficacy: Easy advice & implementable |  |  |  |
|  | Precarious position affecting their perceived vulnerability |  |  |  |

Table 5: Barriers towards other measures across different population subgroups

| **Behaviour: OTHER MEASURES** |  | **Population subgroups** | | | | | | |
| --- | --- | --- | --- | --- | --- | --- | --- | --- |
|  |  | General  Population^[[23]](#footnote-23)^ | People with a migration background ^[[24]](#footnote-24)^ | Young Adults^[[25]](#footnote-25)^ | Low SES^[[26]](#footnote-26)^ | Undocumented Migrant Workers^[[27]](#footnote-27)^ | Older Migrants^[[28]](#footnote-28)^ | Low Health  Literacy^[[29]](#footnote-29)^ |
| **COM-B** | **Barriers** |  |  |  |  |  |  |  |
| **Capability (individual)** |  | | | | | | | |
|  | Occupations not possible to keep distance or work from home |  |  |  |  |  |  |  |
|  | Living situation: many people in small houses |  |  |  |  |  |  |  |
|  | Language: Limited/ no Dutch proficiency |  |  |  |  |  |  |  |
|  | Difficulty to translate measures to individual situations |  |  |  |  |  |  |  |
|  | Effects on mental health |  |  |  |  |  |  |  |
|  | Forgetfulness |  |  |  |  |  |  |  |
| **Opportunity (contextual)** |  | | | | | | | |
| **Physical** | Crowded places |  |  |  |  |  |  |  |
|  | Lack of understanding why the government imposed measures (too complicated) |  |  |  |  |  |  |  |
|  | Conflicting/ inconsistent information |  |  |  |  |  |  |  |
|  | Misinformation |  |  |  |  |  |  |  |
|  | Impracticality/ discomfort (e.g. face masks) |  |  |  |  |  |  |  |

|  |  | **Population subgroups** | | | | | | |
| --- | --- | --- | --- | --- | --- | --- | --- | --- |
|  |  | General  Population | People with a migration background | Young Adults | Low SES | Undocumented Migrant Workers | Older Migrants | Low Health  Literacy |
|  | Limited social interaction |  |  |  |  |  |  |  |
| **Social** | Family obligations/ helping in need (e.g. children) |  |  |  |  |  |  |  |
|  | Stigmatisation & segregation |  |  |  |  |  |  |  |
|  | Specific situations e.g. holidays |  |  |  |  |  |  |  |
|  | Concerns about economy |  |  |  |  |  |  |  |
|  | Strong social norm to keep to cultural and religious practices |  |  |  |  |  |  |  |
|  | Information from own country of origin |  |  |  |  |  |  |  |
| **Motivation (individual)** |  | | | | | | | |
|  | Low risk perception of infection & severity of illness |  |  |  |  |  |  |  |
|  | Ineffectiveness of measures |  |  |  |  |  |  |  |
|  | Boredom |  |  |  |  |  |  |  |
|  | Mild complaints not associated with COVID |  |  |  |  |  |  |  |
|  | Uncertainty/ long duration |  |  |  |  |  |  |  |
|  | Trust in the government |  |  |  |  |  |  |  |
|  | Concerns about privacy and security |  |  |  |  |  |  |  |
|  | Vaccination |  |  |  |  |  |  |  |
|  | Concerns about personal financial situation |  |  |  |  |  |  |  |

Table 6: Drivers towards other measures across different population subgroups

| **Behaviour: OTHER MEASURES** |  | **Population subgroups** | | | | |
| --- | --- | --- | --- | --- | --- | --- |
|  |  | General  Population^[[30]](#footnote-30)^ | Young Adults^[[31]](#footnote-31)^ | Low SES/  Deprived Areas^[[32]](#footnote-32)^ | Undocumented Migrant Workers^[[33]](#footnote-33)^ | Older Migrants^[[34]](#footnote-34)^ |
| **COM-B** | **Drivers** |  |  |  |  |  |
| **Capability (individual)** |  | | | | | |
|  | More knowledge |  |  |  |  |  |
|  | Practical feasibility: advice easy and implementable |  |  |  |  |  |
|  | Following news or looking up information |  |  |  |  |  |
| **Opportunity (contextual)** |  | | | | | |
| **Physical** | Clear communication & information about measures |  |  |  |  |  |
| **Social** | Measures compulsory |  |  |  |  |  |
|  | Penalties for non-compliance |  |  |  |  |  |
|  | Social influences and norms |  |  |  |  |  |
|  | High social participation |  |  |  |  |  |
| **Motivation (individual)** |  | | | | | |
|  | Self-health protection |  |  |  |  |  |
|  | High risk perception |  |  |  |  |  |
|  | Effectiveness of measures (see the added value for themselves and others) |  |  |  |  |  |
|  | Anxiety about COVID-19 |  |  |  |  |  |
|  | Trust in government |  |  |  |  |  |
|  | Perceived susceptibility for COVID-19 |  |  |  |  |  |
|  | Precarious position in society |  |  |  |  |  |
|  | Experiencing serious complaints |  |  |  |  |  |
|  | High impersonal risk perception |  |  |  |  |  |
|  | Protect others/ solidarity |  |  |  |  |  |
|  | High affective response (e.g. worry) |  |  |  |  |  |

1. Bochove et al., 2021, de Vries et al., 2022a, de Vries et al., 2022b, Engbersen et al., 2021, Gebrekrstos., 2022, I&O Research, 2022, IPSOS, 2021, Mouter et al., 2020, Mouter et al., 2022, Sanders et al., 2021, Vader et al., 2022, Yousuf et al., 2021, RIVM, 2022f; RIVM, 2021h, RIVM, 2022e; RIVM, 2021c, RIVM, 2021d; RIVM, 2022d, RIVM, 2021f [↑](#footnote-ref-1)
2. Stichting Alexander, 2021, te Brinke et al., 2021, Vollmann & Salewski, 2021, Euser et al., 2022; RIVM, 2021^e^, Hilverda & Vollmann 2021, Wismans et al. 2021, 2022 [↑](#footnote-ref-2)
3. Antwi-Berko et al., 2022, Stronks et al., 2021, RIVM, 2021g [↑](#footnote-ref-3)
4. Merkelbach et al. 2022 [↑](#footnote-ref-4)
5. el Fakiri et al., 2022, GGD Amsterdam, 2021 [↑](#footnote-ref-5)
6. van Loenen & van den Muijsenbergh, 2022 [↑](#footnote-ref-6)
7. Knottnerus et al., 2021 [↑](#footnote-ref-7)
8. Bochove et al., 2021, de Vries et al., 2022a, de Vries et al., 2022b, Engbersen et al., 2021, Gebrekrstos, 2022, I&O Research, 2022, IPSOS, 2021, Mouter et al., 2020, Mouter et al., 2022, Sanders et al., 2021, Vader et al., 2022, Yousuf et al., 2021 , RIVM, 2022f; RIVM, 2021h, RIVM, 2022e; RIVM, 2021c, RIVM, 2021d; RIVM, 2022d, RIVM, 2021f [↑](#footnote-ref-8)
9. Stichting Alexander, 2021, te Brinke et al., 2021, Vollmann & Salewski, 2021, Euser et al., 2022; RIVM, 2021^e^, Hilverda & Vollmann 2021, Wismans et al. 2021, 2022 [↑](#footnote-ref-9)
10. Antwi-Berko et al., 2022’ Stronks et al., 2021, RIVM, 2021g [↑](#footnote-ref-10)
11. el Fakiri et al., 2022, GGD Amsterdam, 2021 [↑](#footnote-ref-11)
12. van Loenen & van den Muijsenbergh, 2022 [↑](#footnote-ref-12)
13. RIVM, 2022a; RIVM, 2022b; RIVM, 2021a; RIVM, 2021b; CBS, 2021a; RIVM, 2020b [↑](#footnote-ref-13)
14. Stronks et al., 2021 [↑](#footnote-ref-14)
15. Knottnerus et al., 2022 [↑](#footnote-ref-15)
16. de Ruiter et al, 2022 [↑](#footnote-ref-16)
17. Torensma et al., 2021, van den Muijsenbergh et al., 2022 [↑](#footnote-ref-17)
18. GGD Amsterdam, 2021, El Fakiri et al., 2021 [↑](#footnote-ref-18)
19. Mevissen et al., (2022, unpublished manuscript) [↑](#footnote-ref-19)
20. RIVM, 2022a; RIVM, 2022b; RIVM, 2021a; RIVM, 2021b; CBS, 2021a; RIVM, 2020b [↑](#footnote-ref-20)
21. van den Muijsenbergh et al., 2022 [↑](#footnote-ref-21)
22. Mevissen et al., (2022, unpublished manuscript) [↑](#footnote-ref-22)
23. Verberk et al, 2021; Liebst et al., 2020; RIVM, 2020c; RIVM, 2020d, RIVM, 2020b, Hoeben et al.,2021; RIVM, 2020a [↑](#footnote-ref-23)
24. Bakuri et al., 2022; Stronks et al., 2021; Torensma et al., 2021 [↑](#footnote-ref-24)
25. Kollmann et al., 2022, Koning et al., 2022 [↑](#footnote-ref-25)
26. van Loenen et al., 2020 [↑](#footnote-ref-26)
27. van den Muijsenbergh et al., 2022 [↑](#footnote-ref-27)
28. GGD Amsterdam, 2021, El Fakiri et al., 2021 [↑](#footnote-ref-28)
29. Knottnerus et al., 2021 [↑](#footnote-ref-29)
30. Verberk et al, 2021; Liebst et al., 2020; RIVM, 2020c; RIVM, 2020d, RIVM, 2020b, Hoeben et al.,2021; RIVM, 2021a [↑](#footnote-ref-30)
31. Kollmann et al., 2022, Koning et al., 2022 [↑](#footnote-ref-31)
32. van Loenen et al., 2020 [↑](#footnote-ref-32)
33. van den Muijsenbergh et al., 2022 [↑](#footnote-ref-33)
34. GGD Amsterdam, 2021, El Fakiri et al., 2021 [↑](#footnote-ref-34)
